# Biochar applications for treating potentially toxic elements (PTEs) contaminated soils and water: a review

**DOI:** 10.3389/fbioe.2023.1258483

**Published:** 2023-08-17

**Authors:** Xu Zhang, Guoyan Zou, Huaqiang Chu, Zheng Shen, Yalei Zhang, Mohamed H. H. Abbas, Bader Z. Albogami, Li Zhou, Ahmed A. Abdelhafez

**Affiliations:** ^1^ Eco-Environmental Protection Research Institute, Shanghai Academy of Agricultural Sciences, Shanghai, China; ^2^ State Key Laboratory of Pollution Control and Resource Reuse, College of Environmental Science and Engineering, Tongji University, Shanghai, China; ^3^ Shanghai Engineering Research Centre of Low-Carbon Agriculture, Shanghai, China; ^4^ National Engineering Research Center of Protected Agriculture, Shanghai Engineering Research Center of Protected Agriculture, Tongji University, Shanghai, China; ^5^ Soils and Water Department, Faculty of Agriculture, Soils and Water Department, Benha University, Benha, Egypt; ^6^ Department of Biology, Faculty of Arts and Sciences, Najran University, Najran, Saudi Arabia; ^7^ Soils and Water Department, Faculty of Agriculture, New Valley University, New Valley, Egypt; ^8^ National Committee of Soil Science, Academy of Scientific Research and Technology, Cairo, Egypt

**Keywords:** biochar, soil, water, potentially toxic elements (PTEs), remediation technologies

## Abstract

Environmental pollution with potentially toxic elements (PTEs) has become one of the critical and pressing issues worldwide. Although these pollutants occur naturally in the environment, their concentrations are continuously increasing, probably as a consequence of anthropic activities. They are very toxic even at very low concentrations and hence cause undesirable ecological impacts. Thus, the cleanup of polluted soils and water has become an obligation to ensure the safe handling of the available natural resources. Several remediation technologies can be followed to attain successful remediation, i.e., chemical, physical, and biological procedures; yet many of these techniques are expensive and/or may have negative impacts on the surroundings. Recycling agricultural wastes still represents the most promising economical, safe, and successful approach to achieving a healthy and sustainable environment. Briefly, biochar acts as an efficient biosorbent for many PTEs in soils and waters. Furthermore, biochar can considerably reduce concentrations of herbicides in solutions. This review article explains the main reasons for the increasing levels of potentially toxic elements in the environment and their negative impacts on the ecosystem. Moreover, it briefly describes the advantages and disadvantages of using conventional methods for soil and water remediation then clarifies the reasons for using biochar in the clean-up practice of polluted soils and waters, either solely or in combination with other methods such as phytoremediation and soil washing technologies to attain more efficient remediation protocols for the removal of some PTEs, e.g., Cr and As from soils and water.

## 1 Introduction

Pollution is a global challenge that negatively affects life on Earth (Huang et al., 2019; Chen et al., 2020; Zheng et al., 2020). It is responsible for spreading many diseases and approximately 16% of premature death worldwide (Münzel et al., 2022). Since soil is the main terrestrial ecosystem (Qi et al., 2023) then soil pollution can threaten its biodiversity (Lu et al., 2020). Saving soil is essential to save the whole Earth (Gautam et al., 2023). This may take place via monitoring levels of contaminants in the environment and following up effective remediation routes to attain better environmental conditions.

Many contaminants undergo biodegradation while others are relatively stable in soil and water such as potentially toxic elements (PTEs) (Matin et al., 2020). Thus, these contaminants persist in soils for years (Zhang et al., 2020; Zhong et al., 2020) and can have devastating consequences on human health and the surrounding ecosystem (Gui et al., 2023), particularly on children (Egendorf et al., 2020). A point to note is that PTEs may further have negative impacts on female fertility and reproduction (Rashtian et al., 2019).

Environmental risks related to soil pollutants with PTEs should not be appraised only through soil screening levels but also by assessing their bio-available contents in soil (Galán et al., 2019). Mobile fractions of PTEs find their way to the groundwater (Farid et al., 2020) and transfer long distances via the hydraulic continuity of groundwater over vast areas to reach new lands which are not directly subjected to soil pollutants (Bassouny et al., 2020; Farid et al., 2020). Thus, following effective remediation methods could eliminate further environmental contamination with PTEs (Liu et al., 2020). These procedures include physical and chemical remediation methods, e.g., soil washing, encapsulation, soil replacement electrokinetic methods (Chen et al., 2020), amending soils with iron nanomaterials (Baragaño et al., 2020) or hydroxyapatite (Ibrahim et al., 2020)

Water pollution is also of growing concern (Kumar et al., 2019; Dar and Bhat, 2020) because it is a vital resource for all living organisms (Saini et al., 2020). Its decontamination is a requirement to attain better environmental conditions (Singh et al., 2020) following effective and safe remediation procedures (Sahoo and Swain, 2020), e.g., membrane filtration, reverse osmosis, and chemical precipitation (Saini et al., 2020). In spite of that, many of these methods are expensive (Koffi and Okabe, 2020). Otherwise, introducing low-cost materials of high sorptivity might be the optimum choice for water decontamination (Tauqeer et al., 2020). For example, biochar (Zheng et al., 2020) can effectively remove PTEs from contaminated waters within short time periods (Senthilkumar et al., 2020). Its mode of action is via 1) decreasing the solubility of inorganic pollutant ions in soil (Zheng et al., 2020) and water (Shaheen et al., 2019b) because of its alkaline nature (Shi et al., 2020) and it may also form metal ion-chelators (Naveed et al., 2020) of high solubility (Elshony et al., 2019); 2) binding contaminants with the functional groups of biochar to become less mobile or even immobile (Bandara et al., 2020); 3) increasing glomalin-related soil protein **(**GRSP) content in soil (Dubey et al., 2020) which sustains soil quality and minimizes contaminants transfer from soil to aquatic ecosystems (Wang et al., 2020); and 4) stimulating the activity of soil bacteria (Lévesque et al., 2020), especially endophytes (Waqas et al., 2017), to assist host plants to survive under high levels of organic and inorganic pollutants in soil (He et al., 2020).

More details on the advantages and disadvantages of the conventional physical and chemical remediation techniques that are used in decontaminating soils and waters are discussed further. This review also addresses the feasibility of using biochar as a safe organic resource to remediate contaminated soils and water and possible challenges that may affect PTEs binding with biochar to attain successful remediation procedures.

## 2 Environment

The environment is defined as “the sum of all surroundings, including natural resources and other factors that may affect growth and development of living organisms. It is the place (soils, water, air and food) that needs to be protected and restored.” However, unmanaged handling of the environmental resources has resulted in their contamination with PTEs (Abdelhafez and Li, 2014; Abdelhafez and Li, 2015; Abdelhafez et al., 2016; ElShazly et al., 2019a; ElShazly et al., 2019b; Ali et al., 2023; Farid et al., 2023).

### 2.1 Environmental contamination with PTEs

The term “environmental contamination” signifies the existence of unwanted constituents (contaminants) of any type from industrial, municipal, and agricultural wastes in the natural environment (Katayama et al., 2010). They usually originate from anthropogenic sources. Heavy metal “is a general collective term, which refers to the group of metals and metalloids of atomic density greater than 4,000 kg m^-3^, or in other terms their densities are five times more than water” (Nagajyoti et al., 2010). These contaminants are not biodegradable and thus adversely affect the environment (Jinping et al., 2010; Abbas and Abdelhafez, 2013; Abdelhafez and Li, 2014). Generally, most heavy metals are non-essential, e.g., Pb, Cd, Cr, Hg, and As while others, e.g., Fe, Cu, and Zn, are essential for several organisms (known as trace elements). Thus, the term “heavy metals” is vague and meaningless with no chemical or toxicological basis (Duffus, 2002). Alternatively, the term “Potentially Toxic Elements, PTEs” is in use, which is applicable only to the non-essential elements, e.g., Pb and Cd (Nagajyoti et al., 2010).

### 2.2 Sources of contamination with PTEs

The major sources of environmental pollution are probably anthropogenic activities that result from unmanaged practices (Yaron et al., 2012; Abdelhafez and Li, 2014).

#### 2.2.1 Natural sources of PTEs

During rock weathering, many contaminants find their way to surface water and/or groundwater hence possessing potential threats to the surroundings (Ma et al., 2019).

#### 2.2.2 Agricultural practices and PTEs

Agricultural agrochemicals for fertilization and pesticides are widely used worldwide in food production (Abdelhafez et al., 2012) to satisfy the needs of the growing population (Abbas and Meharg, 2008; Abdelhafez et al., 2012; Eid et al., 2019; Mohamed et al., 2019; Abdelhafez et al., 2021). These agrochemicals contaminate agricultural soils with PTEs (Nagajyoti et al., 2010), representing potential ecological risk factors. Likewise, organic fertilizers such as animal manures and sewage sludge enrich soils with Mn, Zn, Cu, Co, Cr, Pb, Ni, and Cd upon their extensive use as fertilizers or amendments (Verklejim, 1993).

#### 2.2.3 Industrial sources of PTEs

Rapid urbanization and industrialization, particularly in developing countries discharge PTEs into rivers and soils. These effluents may change the physical, chemical, and biological conditions of water bodies (Sangodoyin, 1991) while increasing the potential risk associated with using these waters. In the Jinxi River in China, anthropogenic activities were the major source of contamination of water streams with PTEs (Abdelhafez and Li, 2014; Abdelhafez and Li, 2015). Table 1 shows the abundance of metals in effluents from different industrial activities (Abdelhafez et al., 2009; Abdelhafez et al., 2010).

**TABLE 1:** Occurrence of metals or their compounds in effluents from various industries.

Industry	Metal													
	Al	Ag	As	Cd	Co	Cr	Cu	Fe	Hg	Mn	Mo	Pb	Ni	Zn
Mining operations and ore processing	**×**	**-**	**×**	**×**	**-**	**-**	**-**	**-**	**×**	**×**	**×**	**×**	**-**	**-**
Metallurgy and electroplating	**-**	**×**	**×**	**×**	**-**	**×**	**×**	**-**	**×**	**-**	**-**	**×**	**×**	**×**
Chemical industries	**×**	**-**	**×**	**×**	**-**	**×**	**×**	**×**	**×**	**-**	**-**	**×**	**-**	**×**
Dyes and pigments	**×**	**-**	**×**	**×**	**-**	**-**	**×**	**×**	**-**	**-**	**-**	**×**	**-**	**-**
Ink manufacturing	**-**	**-**		**-**	**×**	**-**	**×**	**×**	**×**	**-**	**-**	**-**	**×**	**-**
Pottery and porcelain	**-**	**-**	**×**	**-**	**-**	**×**	**-**	**-**	**-**	**-**	**-**	**-**	**-**	**-**
Alloys	**-**	**-**		**-**	**-**	**-**	**-**	**-**	**-**	**-**	**-**	**×**	**-**	**-**
Print	**-**	**-**		**-**	**-**	**-**	**-**	**-**	**-**	**-**	**-**	**-**	**-**	**×**
Photography	**-**	**×**		**-**	**-**	**×**	**-**	**-**	**-**	**-**	**-**	**×**	**-**	**-**
Glass	**-**	**-**	**×**	**×**	**×**	**-**	**-**	**-**	**-**	**-**	**-**	**-**	**×**	**-**
Paper mills	**×**	**-**		**-**	**-**	**×**	**×**	**-**	**×**	**-**	**-**	**×**	**-**	**-**
Leather training	**×**	**-**	**×**	**-**	**-**	**×**	**×**	**×**	**×**	**-**	**-**	**-**	**-**	**×**
Pharmaceuticals	**×**	**-**		**-**	**-**	**-**	**×**	**×**	**×**	**-**	**-**	**-**	**-**	**-**
Textiles	**×**	**-**	**×**	**×**	**-**	**-**	**×**	**×**	**×**	**-**	**-**	**-**	**×**	**-**
Nuclear technology	**-**	**-**		**×**	**-**	**-**	**-**	**-**	**-**	**-**	**-**	**-**	**-**	**-**
Fertilizers	**×**	**-**	**×**	**×**	**-**	**×**	**×**	**×**	**×**	**-**	**×**	**×**	**×**	**×**
Chlor alkali production	**×**	**-**	**×**	**×**	**-**	**×**	**-**	**×**	**×**	**-**	**×**	**×**	**-**	**×**
Wood preservations	**-**	**-**	**×**	**-**	**-**	**×**	**×**	**-**	**-**	**-**	**-**	**-**	**-**	**-**
Petroleum refining	**×**	**–**	**×**	**×**	**-**	**×**	**-**	**×**	**×**	**-**	**-**	**-**	**×**	**×**

Data obtained from Nagajyoti et al. (2010); Abdelhafez et al. (2012) and Abdelhafez et al. (2016).

Other activities such as mining, refining, smelting, and metal grinding may bring considerable concentrations of PTEs to the surrounding environment (Herawati et al., 2000; Yanqun et al., 2005; Abdelhafez et al., 2016; Mohamed et al., 2018).

Metal ions may be emitted into the atmosphere in the forms of particulates and vapor when subjected to high temperatures and then react with water vapors forming aerosols which finally find their way to soil and water through dry deposition (dispersion by wind) or wet deposition (precipitated in rainfall). In shooting range and smelting operation soils, the levels of Pb sometimes exceeded 1% (Abdelhafez et al., 2014; Abdelhafez et al., 2016).

#### 2.2.4 Soil pollution in relation to domestic and industrial effluents

Many water streams have become contaminated with PTEs via the discharge of industrial and domestic wastes. These contaminants find their way to the topsoil of the surrounding arable lands. Once they come in contact with soil particles, they become sorbed and this process is controlled by diffusion (Abbas and Bassouny, 2018). Considerable amounts of PTEs may go deeper into the soil through common agricultural practices, e.g., plowing and tillage (Hashim et al., 2017). Moreover, hydraulic continuity that exists between ground waters transfers contaminants to locations not directly irrigated with wastewater (Farid et al., 2020).

#### 2.2.5 Aerosols and PTEs

Tiny solid or liquid particles suspended in the Earth’s atmosphere are known as aerosols (Seinfeld and Pandis, 2016). Generally, aerosols are of special importance on a global scale. In this concern, volcanic eruptions are a geothermal source of atmospheric contamination (Gudmundsson et al., 2019). The transportation and deposition of these aerosols increase the potentiality of PTE dispersion in the environment (Soltani et al., 2017). The transmitted fine particulates may be blown over a great distance and accelerated by downpours or snowfall (Behera et al., 2015; ElShazly et al., 2019a).

#### 2.2.6 Other sources of environmental pollution with PTEs

Burning, landfills, incineration, and transportation (automobiles, diesel-powered vehicles, and aircraft) are additional sources of environmental pollution that add Cd, Co, Zn, Cr, Cu, Pb, Hg, Mn, Ni, Al, Fe, and Ti to the environment (Verklejim, 1993; Al-Hiyaly et al., 1998; Hashim et al., 2017). Chromated copper arsenate (CCA) treated wood structures are another source of PTEs when CCA is used as a wood preservative against bacteria, fungi, and termites (Abdelhafez et al., 2009; Abdelhafez et al., 2010).

## 3 Plant response to PTEs

Plants stop growing or even die when grown on soils highly contaminated with PTEs. High levels of PTEs increase the formation of free radicals and reactive oxygen species that cause oxidative stress and cellular damage in plants (Goyal et al., 2020). To survive under such stressful conditions, plants secrete low molecular mass substances such as organic acids and glutathione that bind with PTEs and lessen their mobility in soil. Also, pectin in plant cell walls limits PTE absorption by plants (Feng et al., 2021). Once contaminants enter plant cells, they become sequestered within cellular compartments such as vacuoles and limit their translocation to areal plant parts (Goyal et al., 2020). Tolerant or even hyperaccumulator plants display further mechanisms for controlling these contaminants, nevertheless, they exhibit very slow growth rates and small biomasses (Khan, 2020). Instead, using plant growth-promoting bacteria and mycorrhizae can further improve plant-based remediation strategies (Khan, 2020). Bacteria such as *Alcaligenes faecalis*, *Bacillus cereus,* and *A. faecalis* (Zainab et al., 2021) stimulate the activities of anti-oxidative enzymes such as catalase, peroxidase, and superoxide dismutase (El-Meihy et al., 2019) which scavenge reactive oxygen species (Kaur et al., 2021) and thus help plants to cope with PTE stress and enhance plant growth (Zainab et al., 2021). Non-enzymatic antioxidants, e.g., ascorbate, and metal-binding peptides may also help to lessen metal toxicity within plants (Kaur et al., 2021). Mycorrhizae also retain contaminants in roots and decrease their translocation within plants (Adeyemi et al., 2021).

Phytohormones are chemical messengers that sustain plant growth under PTE stress (Sytar et al., 2019). For example, indole acetic acid (IAA) increases energy trapping capacity in photosystem II (PSII) reaction centers (Ouzounidou and Ilias, 2005). Salicylic acid decreases the levels of free oxygen radicals while increasing plant chlorophyll content (Sytar et al., 2019).

## 4 Impact of PTEs on human health

### 4.1 PTEs exposure pathways

**FIGURE 1:**
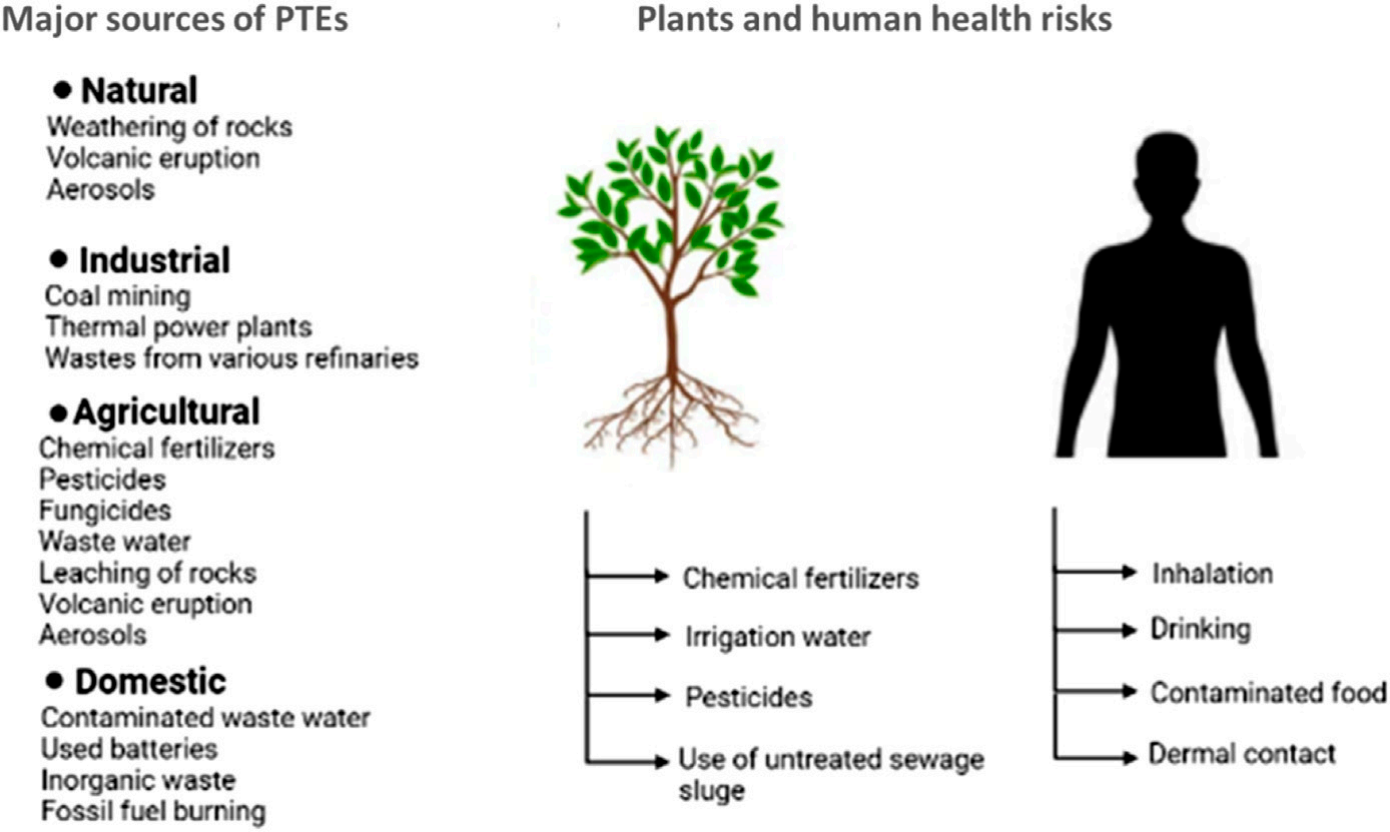
Schematic diagram of PTEs exposure routes.

Humans are exposed to PTEs through different routes: i) ingestion (oral), which includes drinking water, intake of fruit, vegetables, meat and dairy products, and fish and shellfish; ii) inhalation of dust and chemicals volatilized in the air; and iii) dermal contact between human skin and chemicals or soil (Abdelhafez and Li, 2015; Megido et al., 2017). According to Chan et al. (1995), PTEs transmit to humans mainly through inhalation and ingestion routes.Figure 1.

Ingestion is a common exposure route to PTEs (Abdelhafez et al., 2012; Abdelhafez and Li, 2015). It is worth noting that previous studies did not include the distribution pattern of PTEs within the fine fractions of agricultural soil, which presents potential hazards for human health. In this context, fine soil particles of diameters of 10 or 2.5 µm may adhere easily to the skin, carrying PTEs to the human body (Madrid et al., 2008; Kong et al., 2012; Abdelhafez and Li, 2015). These contaminants settle in the higher respiratory tract and the alveolar areas of the lungs (Ajmone-Marsana et al., 2008).

### 4.2 Health effects of PTEs on human health

When these contaminants enter the food chain they have negative implications even at very low levels (Memon and Schröder, 2009). For more details see Table 2. The most problematic PTEs for human health are As, Cr, Cd, Cu, Pb, Zn, Cu, Hg, and Sn (Ghosh, 2010). In particular, As and Cr cause cancer, and Cd, Pb, and Ni lead to kidney failure and other symptoms (Kurniawan et al., 2006; Tripathi et al., 2007; Abbas and Bassouny, 2018). Accordingly, proper remediation protocols should be followed to improve and sustain the environment.

**TABLE 2:** Harmful effects of some PTEs on human health.

PTEs	Harmful effect	References
As	Carcinogenic and interferes with essential cellular processes such as oxidative phosphorylation and ATP synthesis	Tripathi et al. (2007)
Cd	Kidney damage, renal disorder, Itai-Itai (excruciating pain in the bone), hepatic damage, cancer, and hypertension	Kurniawan et al. (2006)
Cr	Carcinogenic, hair loss and has an adverse potential to modify the DNA transcription process	Vilar et al. (2007)
Pb	Renal failure; increased risk for development of cardiovascular disease, encephalopathy, seizures and intellectual disability	Padmavathiamma and Li (2007)
Ni	Dermatitis, nausea, chronic asthma, coughing, bronchial hemorrhage, gastrointestinal distress, weakness and dizziness	Dahiya et al. (2008)
Cu	Brain, liver and kidney damage, insomnia	Kurniawan et al. (2006)
Zn	High dosages can cause dizziness and fatigue	Plum et al. (2010)

## 5 Remediation technologies of PTEs-contaminated water and soils

### 5.1 Remediation technologies of PTEs-contaminated water

There are several remediation protocols that can be followed for decontaminating wastewater, i.e., chemical (chemical precipitation and ion exchange and adsorption), physical (filtration and clarification), and biological (biosorption, biodegradation, and phytoremediation) remediation technologies. These techniques should be applied before water disposal from industries and municipalities into the surrounding environment.

#### 5.1.1 Chemical remediation

Chemical precipitation protocols are broadly utilized for decontaminating wastewater containing high levels of PTEs. These procedures change the soluble contaminants into insoluble forms, thereby enabling their subsequent removal from the liquid phase by physical means, such as clarification and filtration (Arora et al., 2008). For instance, coagulants and flocculants enable the formation of particulate-sized aggregates, and their quantities depend on the pH and alkalinity of the treated water (Nomanbhay and Palanisamy, 2005). Granulated lime and calcium carbonate are efficient coagulants for the removal of As, Ni, Zn, and Cd from groundwater (Song et al., 2005; Lee et al., 2007). In addition, clay minerals can be used effectively to decontaminate aqueous solutions (ElShazly et al., 2019b).

Surface functional groups play an important role in removing metal ions from water by using specific sorbent materials. Table 3 shows some of these functional groups. Herein, more natural and artificial biosorbent materials are examined as adsorbents for the removal of different PTEs from aqueous solutions. Table 4 presents the adsorbent capacities of different biosorbents for PTEs. The adsorption efficiency depends on the pH, sorbent dosage, contact time, temperature, and concentration of metal ions (Abdelhafez and Li, 2016; ElShazly et al., 2019b). Under low pH value, H^+^ competes with metal ions on surface functional groups of the sorbent material hence the removal efficiencies of metal ions decrease considerably (Arief et al., 2008).

**TABLE 3:** Surface functional groups found in different biomasses.

Biomass		Surface functional group	Wavenumber (cm^-1^)		References
Sugar can and orange peel biochars		C-OH stretch	3,448 and 3,430		Abdelhafez and Li (2016)	
		C=O stretch	1637			
		C-C stretch	1384			
		C-O stretch	1101			
		C-OH stretch	1101			
Green taro		OH stretch	3,763		Elangovan et al. (2008)	
		NH2 stretch	2325			
		Several bands from overtone and combination	1920			
		C=O stretch	1707, 1624			
		Ring stretch	1487			
		Antisym stretch	1404			
		C–O stretch	1281			
		SO3 stretch	1184			
		C–O stretch	1019			
		C–CO–C bend	655			
Lignin		Stretching vibrations of aromatic and aliphatic OH groups	3,412		Guo et al. (2008)	
		C–H stretching	2925, 2849			
		Carboxyl and carbonyl stretching	1703, 1648			
		Aromatic skeletal vibrations	1600, 1514, 1425			
		Aromatic methyl group vibrations	1463			
		C–O stretching	1329, 1217			
		Syringyl units	1114, 827			
Olive solid residue		ɣ(O –H)	3,400		Salem and Allia (2008)	
		ɣ(C –H)	2900			
		ɣ(–NH)	1500			
		ɣ(C=C)	1700			
		ɣ(COO–,C=O)	1037			
Sawdust from *Arundo donax*		–OH group	3,600–3,000		Cukierman (2007)	
		C– O, C–C and C–OH bonds	1000–1300			
Seed hulls		–OH group	3,600–3,000			
		C– O, C–C and C–OH bonds	1000–1300			
Sour orange residue		–OH groups	3,423		Khormaei et al. (2007)	
		CH stretching	2925.88			
		C=O band	1631			
		C–O carboxyl band	1257–1244			
Sugarcane bagasse		–OH group	3,600–3,000		Cukierman (2007)	
		C– O, C–C and C–OH bonds	1000–1300			

**TABLE 4:** Adsorption capacity of heavy metals by using different sorbents.

Biosorbent	Metal	pH	T, (°C)	Initial concentration, (mg L^–1^)	Adsorption capacity mg g^-1^	References
Arca shell	Pb(II)	1–7	25 ± 2	10–500	NA	Dahiya et al. (2008)
	Cu(II)					
	Ni(II)					
	Co(II)					
	Cs(I)					
Cactus leaves	Cr(VI)	1–10	30	20–1000	NA	Yuncu et al. (2006)
Crab shell	Cu(II)	3.5–6	NA	500–2000	243.9	Vijayaraghavan et al. (2006)
	Co(II)				322.6	
Exhausted coffee	Cu(II)	5.2	20 ± 1	5–300	11.6	Eseudero et al. (2008)
	Ni(II)				7.25	
Grape stalk	Cu(II)	5.2	20 ± 1	5–300	42.92	
	Ni(II)				38.31	
Maize bran	Cr(VI)	1.4–8	20–40	20–300	NA	Hasan et al. (2008)
Treated sour orange residue	Cu(II)	4.5	28	300	52.08	Khormaei et al. (2007)
						
Orange peel	Pb(II)	1–7	NA	103.5–2070	NA	Xuan et al. (2006)
Palm kernel fiber	Pb(II)	3–8	36 ± 3	120	NA	Ho and Ofomaja (2006)
Tea waste	Cr(VI)	2–5	25–60	50–400	54.65	Malkoc and Nuhoglu (2007)
Ulva lactuca	Pb(II)	2–8	20–50	10–400	34.7	Sari and Tuzen (2008)
	Cd(II)				29.2	
Dairy manure biochar	Cu(II)	NA	NA	63.53–317.7	48.4–54.4	Xu et al. (2013)
	Zn(II)			65.38–326.9	31.6–32.8	
	Cd(II)			112.41–562.05	31.9–51.4	
Crop straw biochar	Cu(II)	NA	25 ± 1	773.36	NA	Tong and Xu (2013)

#### 5.1.2 Physical remediation

Water decontamination can take place via using filtration, air stripping, granular activated carbon absorption, or their combination (Wilson and Clarke, 1993). However, more attention should be paid when using washing technology to remove PTEs due to the leachability of major nutrients (N, P, and K).

#### 5.1.3 Biological remediation

The use of biological remediation technologies is thought to be the optimum tool for remediating contaminated waters/soils.

**FIGURE 2:**
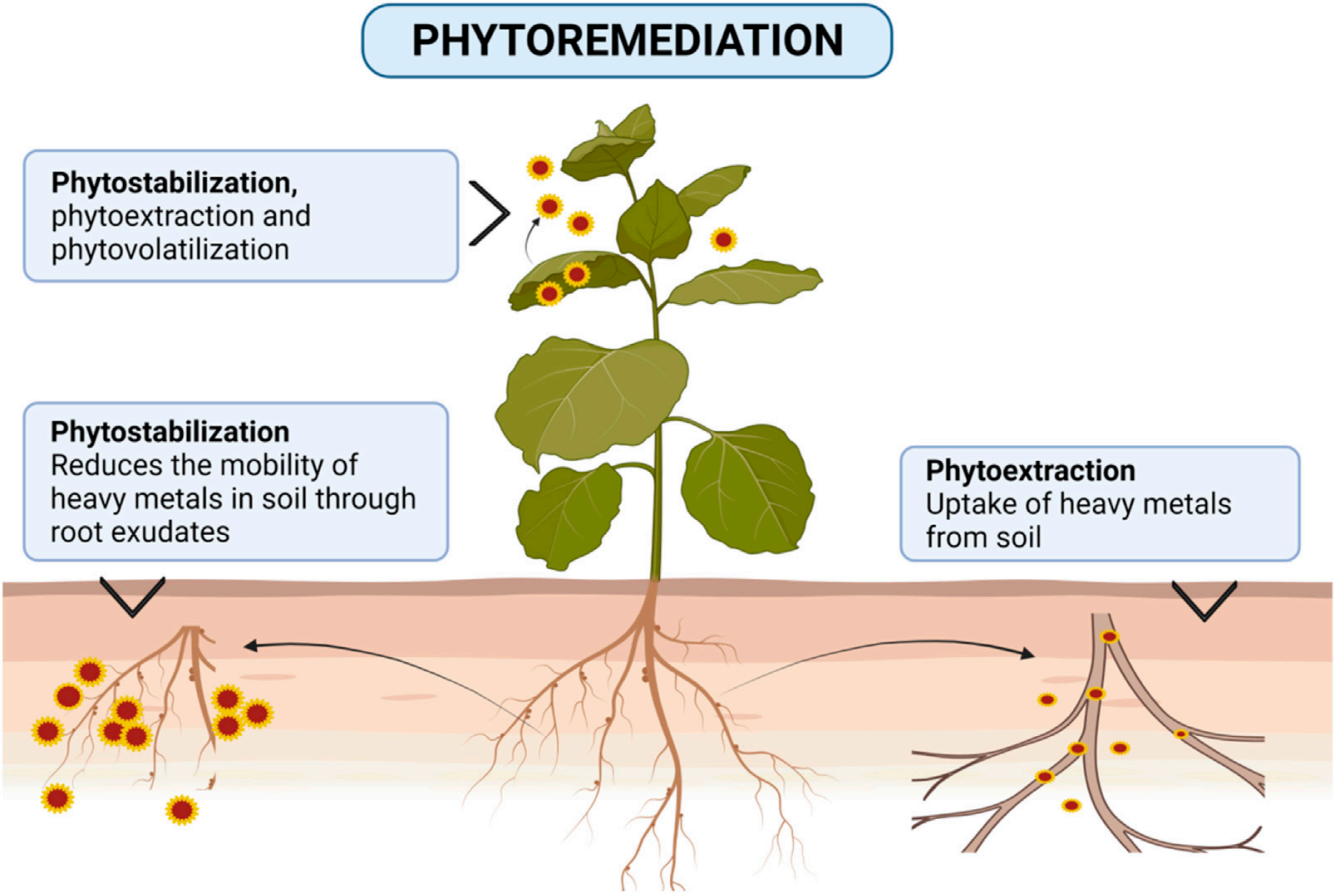
Schematic diagrams of main techniques/strategies of phytoremediation in water and soil.

In this regard, the use of bacteria, fungi, and algae is economical, eco-friendly, and gives good results (Valls and Lorenzo, 2002). These microbes remove contaminants from water in their bodies (Ozdemir et al., 2003; Zouboulis et al., 2004; Congeevaram et al., 2007). Also, plant-induced phytoremediation can degrade or eliminate PTEs in contaminated water/soil. Phytoremediation exploits the plant’s innate biological mechanisms for removing PTEs or eliminates its adverse effects through different mechanisms (Ghosh and Singh, 2005) (Figure 2) as follows.

i) Phytoextraction: the ability to grow plants to absorb and accumulate toxic metals from water
ii) Phytovolatilization: evaporating certain metals through the above-ground parts of the plant
iii) Rhizofiltration: the use of plant roots to remove PTEs from contaminated waters.

### 5.2 Soil remediation technologies

Soil remediation is performed to achieve one of the following goals: 1) removal/extraction of the PTEs from contaminated soils by electrokinetic and/or washing procedures, which is an expensive procedure and might not be applicable for decontaminating vast areas of contaminated soils (Ko et al., 2006; Dermont et al., 2008) or 2) reducing metal mobility with “*in situ*” technologies such as stabilization by different amendments (organic or inorganic) (Chen et al., 2006; Sunarso and Ismadji, 2009) but the contaminants still exist in the soil. Overall, *in situ* soil remediation technologies are directed toward reducing the risk of PTEs in soils and can be classified into four main categories.

#### 5.2.1 Excavation

Excavation is the oldest remediation technology for decontaminating soils, in which contaminated soil layers are replaced by clean ones (Lanphear et al., 2003). However, this method leads to the transfer of contaminants from one place to another, the spread of dust particles, and the transport of contaminated soil to other regions. As a matter of fact, excavation is considered the most expensive method of soil remediation (Lambert et al., 2014; González-Martínez et al., 2019).

#### 5.2.2 Soil washing

Soil washing is a common technique for remediating soils contaminated with PTEs (Khan et al., 2004) in the presence of synthetic complexing agents, using chelators such as ethylene di amine tetra acetic acid (EDTA) and nitrilotriacetate (NTA) to enhance further removal efficiencies of soil contaminants (Arwidsson et al., 2010). However, the low decomposition rates of chelators in soil may cause toxicity and stress to soil biota (Nowack, 2002).

#### 5.2.3 Phytoremediation

Some plants can take up and accumulate contaminants in their aboveground parts (Ebrahimbabaie et al., 2020; Lee et al., 2021), thus limiting their negative consequences to the surroundings (Tusher et al., 2021). This green technology is preferable to other conventional methods because it preserves substrate fertility and, at the same time, reduces the costs of remediation (Riaz et al., 2022). Moreover, it is a suitable eco-friendly solution for remediating large areas, besides being economical (Saxena et al., 2019). The major techniques of phytoremediation are phytostabilization, phytoextraction, and phytovolatilization.

Many plant species have been shown to be efficient in remediating soils and waters contaminated with inorganic (Saxena et al., 2019; Edgar et al., 2021) and organic pollutants (Karaś et al., 2021) (Table 5) yet this process requires long time periods to lessen contaminants to attain acceptable public levels (Mustafa et al., 2022). Adding chelation agents could help in improving the efficiency of this process (Gavrilescu, 2022). Generally, edible crops are not suitable as phytoextractors for potentially toxic elements from contaminated sites (Saxena et al., 2019). Alternatively, aromatic plants can absorb and accumulate high concentrations of PTEs in the harvestable foliage while their oil is free from the risk of PTE accumulation (Lajayar et al., 2017). Also, plants grown for biofuel production are guaranteed for the phytoextraction process of PTEs from soils (Edgar et al., 2021; Rheay et al., 2021).

**TABLE 5:** Phytoextraction results of PTEs from contaminated soils.

Plant species	Contaminant	Its feasibility	References
*Achillea millefolium*	Mercury	Phytovolatilization of Hg may cause public fear	Wang et al. (2012)
*Eupatorium perfoliatum*	Polycyclic aromatic hydrocarbons in soil	Not feasible because of its low bioavailability	Ahn et al. (2005)
Hemp (*Cannabis sativa* L.)	Potentially toxic elements, radionuclides, and organic contaminants and as a feedstock	Feasible for bioenergy production	Rheay et al. (2021)
Ryegrass (*Lolium perenne* L.)	Potentially toxic metals	Washing with chelating agents (HCl, EDTA, and NTA) coupled phytoremediation is feasible for metal-contaminated soil remediation	Xiao et al. (2019)
Maize (*Zea mays*)	Arsenic	Arsenic phytoremediation potential of the maize plants was found to be economical for sandy loam soil with a 1% compost level and for clay loam soil at a 2.5% compost level	Mehmood et al. (2021)
S. alfredii and oilseed rape	Cadmium	Dry weights of S. alfredii and oilseed rape were enhanced under intercropping pattern and decreased the remediation period	Zhang et al. (2021)
Rosularia adenotricha, Catharanthus roseus, Allium griffithianum, Himalaiella heteromalla, *Stellaria media*, *Salvia* moorcroftiana and *Marrubium vulgare*	Chromium	Efficient phytoextractors of Cr from soil	Sajad et al. (2020)
aromatic plants from families—Poaceae, Lamiaceae, Asteraceae, and Geraniaceae	Potentially toxic elements	Feasible for the phytoextraction process	Pandey et al. (2019)

The removal of PTEs from soil takes place by selecting tolerant plants which have the ability to accumulate PTEs within their aboveground tissues (shoots) (Ali et al., 2013), at concentrations exceeding 0.1% for Cu, Cr, Ni, or Pb, or >1% for Mn or Zn (Yoon et al., 2006). PTEs may also be physically stabilized in soil and this method lessens their translocations to areal plant parts (phytostabilization). Otherwise, PTEs can be transformed into a gaseous form via leaves (phytovolatilization). The main mechanisms of the phytoremediation technique for remediating PTEs contaminated soils are shown in Figure 2.

### 5.3 Stabilization/solidification (S/S)

The stabilization/solidification method is used to lessen the solubility of PTEs using non-toxic materials (organic or inorganic) (Chen et al., 2006; Sunarso and Ismadji, 2009; Abdelhafez et al., 2014), especially in land with high contamination levels. Sorption and/or precipitation are the main routes for decreasing PTE bioavailability in soil (Basta and McGowen, 2004). These amendments include organic additives, phosphates, alkaline agents, and biosolids (Table 6).

**TABLE 6:** Some amendments used for the stabilization of heavy metals in contaminated soils.

Amendment	Heavy metal	References
Flyash	Pb	Ciccu et al. (2003)
Cyclonic Ash	Cd, Pb, and Zn	Brown et al. (2005)
Cement and rice husk ash	Pb	Yin et al. (2006)
Phosphate amendment	Pb	Cao et al. (2009)
Phosphogypsum	Pb	Rodríguez-Jorda et al. (2010)
Zeolite	Cd	Lin et al. (1998)
Lime	Cu, Fe, and Zn	Khan and Jones (2009)
Zeolite	Cu and Zn	Fawzy (2008)
Mono calcium phosphate- Calcium carbonate	Cd, Cu, Ni, Pb, and Zn	Wang et al. (2001)
Phosphate rock and phosphoric acid	Zn, Cu, and Pb	Cao et al. (2009)
Biochar derived from Stems of willow	Cd, Cu, Pb, and Zn	Trakal et al. (2011)
Biochar derived from hardwoods	As, Cd, and Zn	Beesley and Marmiroli (2011)

Table 7 shows a comparison between the conventional remediation technologies. Clearly, the stabilization/solidification (S/S) technique seems to be one of the most efficient methods because it is a cost-effective method that has rapid outcomes (USEPA, 2004). It is therefore recognized as the “best demonstrated available technology (BDTA)” by the USEPA for land disposal of most PTEs (Singh and Pant, 2005) in highly contaminated soil. From the aforementioned information, it seems obvious that the reuse of organic wastes is essential to remediate the PTE-contaminated water and soils.

**TABLE 7:** Comparison of conventional remediation technologies of heavy metal contaminated soils.

Remediation technology	Advantages	Disadvantages
Excavation and soil capping (Physical)	-Effective	-High costs
	-Short treatment time	-Loss of highly fertile surface soil
	-Heavy metals were removed permanently from the site	-Generation of dust and vapor during the excavation, which may cause air pollution
		-Groundwater controls may be needed
Soil washing sand flushing (Physical and chemical)<	-Effective	-Less effective when the soil contains high contents of silt, clay, and organic matter
	-Can be done onsite by using portable equipment	-Wastewater generated needs to be treated and residue disposed of
	-The treated soils can be returned again to place	
	-Ability of metal recovery	
	-Highly applicable in coarse soils	
Phytoremediation (Biological)	-Does not require expensive equipment and low costs	-Long time period required
	-The plants can be easily monitored	-Remediation extends only to the depth of the root zone
	-The possibility of the recovery and re-use of valuable metals	-Not effective for highly contaminated soils
		-Climatic conditions are limiting factors
		-Slow growth and low biomass require a long-term commitment
Stabilization/solidification (Chemical)	-Low costs	-Depth of contaminants may limit some types of application processes
	-Time to complete the remediation is relatively short	-The solidified material may affect future uses of soil
		-Treatment needs to be renewed periodically

Data obtained from Behm et al. (1997); Mulligan et al. (2001); Khalid et al. (2017).

## 6 Organic wastes and biochar

Every year, a huge amount of organic waste is produced annually without being properly recycled, especially in developing countries. For example, the amount of sugar cane and orange waste which is produced annually in China is estimated to be 123 and 32.7 million mega-grams (Abdelhafez et al., 2016). The corresponding amounts produced annually in Egypt exceed 44.0 million mega-grams. These residues should be recycled to be used in sustaining the environment rather than polluting it. In particular, biochar is a carbon-rich material product manufactured through pyrolysis of plant residues, i.e., wood or plant leaves at a relatively low temperature (<700°C) in the absence of oxygen or under limited oxygen conditions (Abdelhafez et al., 2014; Lehmann and Joseph, 2015; Abdelhafez et al., 2016; Abdelhafez and Li, 2016; Farid et al., 2022; Khalil et al., 2023).

### 6.1 Biochar for CO_2_ mitigation and improving soil fertility

Biochar has gained significant attention within the last few years because of its positive role in lessening CO_2_ emissions when used as an amendment to improve soil quality (Jeffery et al., 2011; Kookana et al., 2011; Abdelhafez et al., 2014; Abdelhafez et al., 2016). It is thought that biochar significantly reduces the readily available C fraction to microbes, thus, it slightly or insignificantly induces the activities of microbes and soil enzymes. This, in turn, enhances long-term carbon sequestration. Also, the dominance of aromatic organic carbon, which is very stable in the environment, guarantees its long-term existence in soil (Lehmann, 2007; Abdelhafez et al., 2017). For years, extensive human activities have caused degradation in soil quality and fertility. This negatively affects food production in many regions around the world. Accordingly, improving soil characteristics is necessary to overcome the lack of food production, especially in sub-Saharan Africa and South Asia, where the malnutrition percentages ranged from 32% to 22% of the total population, respectively (FAO, 2019). The solution is biochar as it can be used successfully to restore soil fertility and improve the soil’s physical, chemical, and hydrological properties (Novak et al., 2009; Free et al., 2010).

### 6.2 The potentiality of biochar for remediating PTE-contaminated water and soils

The role of biochar in improving soil fertility is not well-identified and is still being intensively studied. Only limited studies have investigated the potentiality of biochar derived from different organic sources in remediating soil and water contaminated with PTEs. Because of its porous structure (Abdelhafez et al., 2017), high cation exchange sites density, and net negative charge (Jing et al., 2019) biochar has a high capability to sorb PTEs (Jing et al., 2019) which diffuse into its micropores (Nguyen et al., 2008). This may further contribute to PTE precipitation in soils (Jing et al., 2019). The stabilization of PTEs in soil owing to biochar application can be attributed to the alkaline nature of biochar (Abdelhafez et al., 2017; Buss et al., 2019) which allows the functional groups of biochar to protonate and dissociate, replacing H^+^ in the solution with cationic PTEs (e.g., Pb and Cd) (Shaheen et al., 2019b). Also, increasing pH decreases the solubility and mobility of PTEs in soil (Shaheen et al., 2019b). With time, the exchangeable forms of PTEs co-precipitate in the form of inner-sphere complexes (Abdelhafez et al., 2016; Abdelhafez et al., 2017; Penido et al., 2019; Yuan et al., 2019) and change into less labile organic and residual fractions (Mohamed et al., 2018; Matin et al., 2020).

Although, this organic source may contain PTEs, the elevated pyrolysis temperature transforms PTEs into more stable and less toxic forms (de Souza et al., 2019). Thus, biochar acts as an efficient biosorbent for PTEs in contaminated soil (Mohamed et al., 2018) and water (Shaheen et al., 2019b). Biochar can also remove high amounts of herbicides from solutions by coating the dissolvable surfaces. It can therefore be used effectively to boost the health and nutrient status of the soil, particularly in the arid calcareous soil. Recent studies have shown the success of utilizing biochars in remediating water and soils contaminated with PTEs (Table 8).

**TABLE 8:** Different types of biochar for the remediation of PTE-polluted soil and water.

Biochar	Media	PTEs	References
Wheat straw	Soil	Zn, Cd	Qian et al. (2019)
Sugar cane	Soil	Pb	Abdelhafez et al. (2016)
Orange peel, sugarcane bagasse	Soil	Pb, As	Abdelhafez et al. (2014)
Rice straw	Soil	Pb, Cu	Wang et al. (2019)
Sugar cane straw	Soil	Zn, Pb, and Cd	Puga et al. (2015)
Orchard prune residue	Soil	Cd, Cr, Cu, Ni, Pb, and Zn	Fellet et al. (2011)
Hardwood	Soil	As,Cd, Cu, and Zn	Beesley and Marmiroli (2011)
Chicken manure and green waste	Soil	Cd, Cu, and Pb	Park et al. (2011)
Chicken manure	Soil	Cr	Choppala et al. (2012)
Sewage sludge	Soil	Cu, Ni, Zn, Cd and Pb	Méndez et al. (2012)
Rice straw	Soil	Cu, Pb, and Cd	Jiang et al. (2012)
Quail litter	Soil	Cd	Suppadit et al. (2012)
Wood and bark	Water	Cd and Pb	Mohan et al. (2007)
Sugar cane bagasse and orange peel	Water	Pb	Abdelhafez and Li (2016)
Dairy manure	Water	Pb	Cao et al. (2009)
Dairy waste and sugar beet	Water	Pb, Cu, Ni, and Cd	Inyang et al. (2012)
Dairy manure	Water	Cu, Zn, and Cd	Xu et al. (2013)
Crop straws	Water	Cu	Tong and Xu (2013)
Digested sludge	Water	Pb and Cd	Ni et al. (2019)
Rice straw	Water	Pb	Shen et al. (2019)
Algae	Water	Co	Bordoloi et al. (2017)

The effect of biochar application on mobilizing metal ions in soil is confusing, for example, Abdelhafez et al. (2010; 2016) found that biochar increased the availability of Cu and As in biochar-treated soil. In addition, Alaboudi et al. (2019) explained that the addition of wood biomass biochar led to the transformation of Cr(III) into Cr(VI) due to increasing soil pH; consequently, its uptake was increased by maize plants. Furthermore, Shaheen et al. (2019a) reported that biochar applications increased the mobility of some PTEs in soil, such as Cu and As, through association with dissolved organic carbon. However, Lomaglio, et al. (2016) found that the addition of biochar decreased the labile concentration of Pb while increasing As and Sb solubility. Therefore, the role of biochar in stabilizing PTEs is still not well understood.

The degree of biochar stability depends mainly on the dose of applied biochar in addition to its mode of action period (Wang et al., 2019). In this regard, microbial and enzymatic activities (dehydrogenase, acidic and alkaline phosphatase, and urease) were higher in soils mixed with aged biochar than in fresh biochar soil (Yadav et al., 2019).

A point to note is that application of biochar not only increases the non-enzymatic antioxidants (soluble phenolic compounds and free proline) that increase plant tolerance to PTE stress (Kumar et al., 2022) but also stimulates the activities of metal-tolerant plant growth promoting rhizobacteria (Zhou et al., 2022) and mycorrhizae (Ortaş, 2016). Moreover, biochar increases plant growth promoting hormones to alleviate salt stress (Farhangi-Abriz and Torabian, 2018). This could be useful to increase the phytohormones which are responsible for alleviating PTE stress in plants.

Thus, future studies are required to investigate the effects of aging (from fresh to old) on the physiochemical properties of biochars in soils (differing in types) under field conditions. In addition, the effect of biochar on PTE solubility, especially, Cr, Cu, and As, is still a matter of concern.

## 7 Feasibility of the biochar/phytoremediation technique as a sustainable approach to manage PTEs polluted soils

Phytoremediation utilizes the natural ability of plants to uptake and accumulate contaminants from the media. Plants can hyperaccumulate PTEs, and certain species have shown remarkable tolerance and efficacy in remediating contaminated soils (Zheng et al., 2020). With the application of biochar, the efficiency of the phytoremediation process increases, e.g., its application enhanced plant growth, and increased metal sequestration. The biochar/phytoremediation technique operates through various mechanisms. Biochar improves soil properties by enhancing water retention, increasing nutrient availability, and stabilizing soil pH (Park et al., 2011). It acts as a sorbent for PTEs, reducing their mobility and bioavailability. In combination with plants, biochar provides a stable environment for root development and facilitates the uptake and translocation of PTEs by plants. It is worth noting that the biochar/phytoremediation technique offers several environmental benefits. It promotes carbon sequestration, as biochar remains longer in soils. This helps mitigate climate change by reducing greenhouse gas emissions. Additionally, the technique minimizes soil erosion, enhances soil fertility, and promotes biodiversity by creating a favorable habitat for soil organisms. Despite its promise, the biochar/phytoremediation technique faces certain challenges. The selection of suitable plant species, biochar properties, and application rates requires careful consideration (Cao et al., 2009). Long-term monitoring is essential to evaluate the persistence of remediation effects. Furthermore, the economic feasibility and scalability of the technique need to be assessed to encourage its widespread implementation.

## 8 Precautions while selecting appropriate remediation technology for PTEs

The selection of the appropriate remediation method is a function of several factors as follows.

i) Soil pH is a very important factor affecting the bioavailability of PTEs which decrease under alkaline conditions (Abdelhafez et al., 2012). In addition, soil texture and organic matter contents play significant roles in this concern, i.e., the higher the fine particles (clay and silt) contents in soil, the harder the metal extraction, since extracted PTEs might be adsorbed by iron-manganese oxides and located on the surfaces of those soil particles (Bradl, 2004). Furthermore, site conditions such as bedrock, large boulders clays, moisture content, and oily patches affect the solidification/stabilization and vitrification remediation technologies (Mulligan et al., 2001).
ii) Types of contaminants to be removed (organic/inorganic): some metals such as arsenic (As), chromium (Cr-VI), and mercury (Hg) do not form hydroxides (less soluble). Therefore, solidification/stabilization seems to not be appropriate for ameliorating soils contaminated with these types of PTEs (Mulligan et al., 2001). Furthermore, the high levels of Pb concentrations in shooting range and metal smelter-contaminated soils, which may exceed 1% (Yanqun et al., 2005; Levonmaki et al., 2006; Hashimoto et al., 2009), decrease the efficiency of remediating such soils by using the phytoremediation approach. The vitrification method is probably more suitable in areas containing low volatile metals with high glass solubility such as Pb, Cr, As, Zn, Cd, and Cu-contaminated soils (Smith et al., 1995). Unlike solid metals, Hg is characterized by its high volatility and low glass solubility, therefore, the vitrification method is unsuitable for remediating Hg-contaminated soils owing to the toxic gasses emitted during the vitrification process (Mulligan et al., 2001).
iii) The end use of contaminated soil: the future use of the soil should be considered before the remediation process to avoid unnecessary expenditures. Ok et al. (2010) showed that the pH of soil increased up to 12.5 when amended by calcined oyster shell powder in order to stabilize Cd and Pb. These types of remediated soils become unsuitable for agricultural purposes due to their high soil pH which limits the availability of nutritive elements.

## 9 Future outlook and conclusion

Potentially toxic metals are released into the environment mainly through anthropogenic activities as well as geological sources. These contaminants are responsible for spreading many diseases and almost 16% of premature deaths worldwide. A number of remediation techniques can therefore be followed to ameliorate PTE-contaminated soil and water, among which the immobilization technique is considered the best approach due to its easy availability and cost-effectiveness. In particular, the immobilization or removal of PTEs from soil and water with biochar has several advantages owing to its specific surface area, porous structure, and high selectivity for all the PTEs. We have reviewed more than 200 articles to compare the efficiency of existing technologies and biochar application in the remediation of contaminated soils and waters. Generally, the major mechanisms involved in PTE binding with biochar are complexation, precipitation, and adsorption.

Biochar acts as an efficient biosorbent for many PTEs in soil and water. It may, however, increase the mobility of other PTEs such as Cu and As via association with dissolved organic carbon. The degree of stability of biochar-PTEs in soil depends on the dose of applied biochar as well as its aging. More research is therefore needed to clarify this relationship in both soil and water. Furthermore, biochar can remove high amounts of herbicides from solutions. Thus, future studies should focus on the role of functional groups of biochar in the PTE remediation process, considering successive applications and long-term field investigations. The combination of different immobilizing agents in improving the phytoremediation efficiency of PTEs with biochar and also their consequences on the growth of plants by adding the required essential elements could be a matter of concern in future research.

Overall, the biochar/phytoremediation technique could have a significant impact as a sustainable approach for managing PTEs-polluted soils Its synergistic effects enhance PTE immobilization, reduce environmental risks, and promote ecosystem restoration. Although challenges exist, ongoing research and technological advancements are expected to address these limitations, further improving the feasibility and effectiveness of this technique.
